# Healthcare providers’ perceptions and understanding of voluntary medical male circumcision in KwaZulu-Natal, South Africa: A qualitative study

**DOI:** 10.4102/safp.v63i1.5318

**Published:** 2021-08-30

**Authors:** Celenkosini T. Nxumalo, Gugu G. Mchunu

**Affiliations:** 1 KwaZulu-Natal Department of Health, Ndwedwe Community Health Centre, Verulam, South Africa; 2 School of Nursing and Public Health, Faculty of Health Sciences, University of KwaZulu-Natal, Durban, South Africa

**Keywords:** HIV prevention, healthcare workers, medical circumcision, uptake, voluntary medical male circumcision

## Abstract

**Background:**

There is compelling evidence that voluntary medical male circumcision (VMMC) reduces the chances of heterosexual transmission of HIV infection. Healthcare workers are among the key influencers in terms of the scale-up of VMMC as they are often involved in mobilisation for uptake. There is a paucity of qualitative research on healthcare workers’ experiences, understanding and perceptions of VMMC; particularly in the South African rural primary healthcare context. This study was conducted to examine healthcare workers perceptions and understanding of VMMC in KwaZulu-Natal, South Africa.

**Methods:**

The study employed a qualitative approach using a phenomenographic design. A purposive sample of 15 doctors, nurses and clinical associates working in 6 different rural clinics in KwaZulu-Natal, South Africa, were interviewed in English in-depth using a semi-structured interview schedule. The interviews were audio-recorded, and transcribed. The results were analysed thematically using phenomenographic data analysis procedures.

**Results:**

Categories of description in participants’ perceptions and understanding of VMMC emerged. The findings of this study revealed that healthcare workers perceptions and understanding of VMMC were predominantly influenced by the hegemonic religious and cultural norms associated with male circumcision in KwaZulu-Natal, South Africa.

**Conclusion:**

The findings of this study suggest that tailored training to address healthcare workers misperceptions and poor understanding of VMMC is necessary to ensure that they become effective custodians for VMMC implementation.

## Introduction

The results of three randomised controlled trials conducted in Kenya, Uganda and South Africa, have proven that voluntary medical male circumcision (VMMC) is an effective biological human immunodeficiency virus (HIV) prevention strategy.^1,2,3^ Following these results, the World Health Organization (WHO) recommends that regions of high HIV prevalence adopt VMMC as an additional HIV prevention intervention.^4^ In 2010, VMMC was introduced in KwaZulu-Natal (KZN), South Africa following recommendations made by the WHO.^5^

Since the roll-out of VMMC, professional healthcare workers have been at the forefront of implementing the service as stipulated by standardised national and international protocols.^6,7^ Adequate clinical training, knowledge and competence are some of the essential elements required to ensure delivery of an efficient and effective VMMC service.^8^ Over the past decade, the implementation of VMMC in KZN, South Africa, was robust, with just over 1.2 million medical circumcisions carried out since 2010. A significant proportion of these medical circumcisions were carried out on boys in the 10–14 year age group, thus allowing for the long-term protection of this age group against HIV infection.^9,10^ However, if the province and the country are to see an immediate reduction in new infections, the target group for VMMC should be males in the 15–49-year age group, as this is the sexually active group.^11^ In instances where the cohort males aged 10–14 years who are now 20–24 years were medically circumcised, that gap in VMMC lies amongst those who are older or those that were not reached in their childhood. This supports the notion of meeting age specific targets for VMMC so that the maximum benefits of partial protection against HIV infection are attained.

Research carried out on the uptake of VMMC has revealed several challenges that hinder the uptake among males. There are individual, social and structural barriers particularly those in the primary target group (15–49-year-old) for medical circumcision, including perceptual factors, the role of social influencers such as peers, key figures and role models, as well as the availability of healthcare services for men.^12,13,14^ The scale-up of VMMC, so that the stipulated age-specific targets are met, is also dependent upon health service providers’ attitudes, perceptions and beliefs about the efficacy of VMMC.

While there has been extensive research on the uptake of VMMC in South Africa and the rest of southern and east Africa, where male hegemony is similarly part of the socio-cultural value system, there is a paucity of contextual research on healthcare workers’ perceptions and understanding of VMMC in southern Africa, particularly in KZN, South Africa. Aside from studies of healthcare workers’ attitudes to and understanding of VMMC prior to the roll-out of VMMC in South Africa,^15^ current research on healthcare workers’ perceptions of VMMC in KZN, South Africa is limited to pharmacy and nursing students, and both of which are in urban settings.^16,17^

The present study examines professional healthcare workers’ perceptions and understanding of VMMC in KZN, South Africa. An awareness of healthcare workers’ perceptions and understanding of VMMC has important policy implications in terms of the education and training of healthcare workers regarding VMMC.

## Research design

A qualitative approach using a phenomenographic study design was used. This study was part of a larger study conducted to analyse primary healthcare stakeholders’ experiences, understanding and conceptualisation of VMMC in KZN, South Africa, so as to propose a relevant intervention to support uptake.

Phenomenography is a qualitative research design that is interpretive in nature and seeks to clarify, discern and analyse the various ways in which individuals conceptualise, understand and experience a phenomenon in the world around them.^18^ It does not consider the subject and aspect of the world as separate entities, but rather the individual’s experience, conceptualisation or understanding is seen as setting up a relation between that person and a given phenomenon in the world.^19^

## Research setting

This study was conducted at six different rural clinics offering VMMC services in KZN, South Africa. The selected clinics serve an estimated population of 110 000–250 000 men in the age group of 15–49 years. Each clinic performs between 700 and 2800 VMMC’s on boys and men annually, with the majority being young male children in the 10–14-year age group.

## Sampling and recruitment

Purposive sampling was used to select the clinics and the participants for data collection. Participants who were directly involved in rendering VMMC services were purposively selected from a study population of nurses, doctors and clinical associates working at the selected clinics.

## Data collection

Individual in-depth interviews were conducted using a semi-structured interview guide that contained a demographic section and guiding questions to elicit respondent perceptions and understanding of VMMC in KZN, South Africa. The interview schedule was written in English and translated into Isizulu. However, all interviews for this study were conducted in English as participants were comfortable with English as the language of communication. An audiotape was used to record all interviews. Each interview was conducted in a private consulting room within each of the clinics and lasted between 20 min and 35 min. Data collection ceased once data saturation was reached.

## Data analysis

Following data collection, the audio recorded data were transcribed verbatim and then analysed following phenomenographic data analysis procedures. The data analysis was an iterative process which followed a step-by-step approach as recommended by Sjöström and Dahlgren.^20^ Firstly, the interview transcripts were read several times while listening to the audiotapes to ensure that data were accurately transcribed, and to gain an overall understanding of the data. The second step entailed a more focused reading so as to extract similarities and differences from the data. Thirdly, significant aspects of the transcript were extracted. The fourth step involved a preliminary grouping of similar responses leading to the creation of an initial list of descriptive categories that were later refined through constant comparison with the transcript. The final set of categories were named and the outcome space was formulated, based on the internal relations between the categories of description.

### Trustworthiness

Data was analysed in collaboration with an expert in qualitative research methods to ensure credibility and confirmability. To ensure richness of the data, a qualitative approach using in-depth interviews was employed and initial data analysis was conducted concurrently with data collection which ceased once data was saturated. To ensure credibility and dependability, voice recordings were reviewed many times and compared with the transcribed data. A detailed methodology of the study is provided to ensure transferability.

### Ethical considerations

This study was part of a larger study conducted to analyse the qualitative differences in primary healthcare stakeholders’ experiences, understanding and conceptualisation of VMMC in KwaZulu-Natal, South Africa. Ethical approval to conduct this study was obtained from the Biomedical Research Ethics Committee of the University of KwaZulu-Natal (BE 627/18). Informed consent was obtained verbally and in writing from the participants prior to data collection. A written information sheet was provided to the participants clarifying the nature of the study, and written consent was sought from the participants after verbal approval was obtained.

## Results

Fifteen healthcare providers took part in the study. The participants included registered nurses (*n* = 5), enrolled nurses (*n* = 3), nursing assistants (*n* = 2) clinical associates (*n* = 3) and medical doctors (*n* = 2). The demographic details of the participants are shown in Table 1.

**TABLE 1:** Profile of participants.

Participant	Age	Gender	Level of education	Designation	Duration involved with VMMC	Religious/cultural beliefs
Partcipant 1	27	Male	University degree	Registered Nurse	2 years	New testament Christian
Participant 2	34	Male	College diploma	Registered Nurse	3 years	Old testament Christian
Participant 3	31	Male	College diploma	Registered Nurse	3 years	Old testament Christian
Participant 4	42	Male	College diploma	Registered Nurse	5 years	African traditional religion
Participant 5	38	Female	College diploma	Registered Nurse	18 months	New testament Christian
Participant 6	29	Male	Higher certificate	Enrolled Nurse	1 year	New testament Christian
Participant 7	33	Male	Higher certificate	Enrolled Nurse	3 years	African traditional religion
Participant 8	29	Female	Higher certificate	Enrolled Nurse	4 years	New testament Christian
Participant 9	28	Male	Certificate	Nursing assistant	2 years	New testament Christian
Participant 10	34	Male	Certificate	Nursing assistant	5 years	Old testament Christian
Participant 11	39	Male	University degree	Medical doctor	4 years	Old testament Christian
Participant 12	34	Male	University degree	Medical doctor	6 years	New testament Christian
Participant 13	23	Male	University Junior degree	Clinical associate	18 months	New testament Christian
Participant 14	26	Male	University junior degree	Clinical associate	14 months	African traditional religion
Participant 15	22	Male	University junior degree	Clinical associate	8 months	New testament Christian

VMMC, voluntary medical male circumcision.

### Perceptions of voluntary medical male circumcision in KwaZulu-Natal, South Africa

Three main categories of participants’ perceptions emerged from the data analysis. These categories are described below, and relevant supporting statements from the participants are provided.

#### Foreskin removal

Healthcare workers perceived VMMC to be the removal of the foreskin of the penis but the extent of foreskin removal was not explicitly mentioned. This removal was seen to be medically beneficial. The following statements serve to justify this perception:

‘A surgical procedure where the foreskin of the male genitalia is removed as it is a good medium of transmission for HIV and STI’s [*sexually transmitted infections*].’ (Participant 3, male, 31 years, registered nurse)

‘Medical male circumcision is the removal of the foreskin, a small piece of the skin. They do not remove the whole thing. This is to help prevent contracting any diseases. You are ess susceptible to diseases if you are circumcised.’ (Participant 4, male, 34 years, registered nurse)

‘In short I would say it is removal of the foreskin covering the glans. It’s so that the glans is exposed.’ (Participant 2, male, 34 years, registered nurse)

#### A procedure for young boys

Although the healthcare workers in this study appeared to be aware that medical circumcision is for men of all ages, they thought the procedure should mainly be performed on males at a young age. This was stated in their descriptions of the best time to perform medical circumcision. The excerpts below serve to support this category:

‘For convenience, I think it is better circumcising boys starting from eight years and above … Here we do them when they are that age because they are able to tolerate anaesthesia.’ (Participant 4, male, 34 years, registered nurse)

‘The older generation of boys and men are a problem because they often have adhesions that are a challenge when doing circumcision. In boys of a very small age, there are also challenges so I think 12 years is okay.’ (Participant 1, male, 27 years, registered nurse)

‘It is better to do the procedure on boys from 12 years old because they understand and know everything better. When you explain things to them, they do not have a problem only when you inject them there is some bit of challenge but after that they are fine and you carry on.’ (Participant 5, female, 38 years, registered nurse)

#### Service to be rendered by males

Healthcare providers views are influenced by their participation in and knowledge of a socio-cultural value system where circumcision is associated with traditional gender specific rites of passage. The following statements support this category:

‘In my opinion, I can say that sometimes you do not become comfortable to be assisted by a female.’ (Participant 7, male, 33 years, enrolled nurse)

‘In the rendering services, I think it best for a man to be performing the circumcision seeing that the procedure is very much influenced [*by*] traditional beliefs especially here in the Zulu culture. Even more this is something private, for a woman to be involved seems sort of disrespectful to a man and may cause him to not want to get medically circumcised.’ (Participant 4, male, 42 years, registered nurse)

‘In terms of performing the procedure, it must be males.’ (Participant 14, male, 26 years, clinical associate)

### Understanding of voluntary medical male circumcision in KwaZulu-Natal, South Africa

Three main categories of participants’ understanding regarding VMMC emerged from the data analysis. The categories of description and relevant support statements from the participants are described below.

#### Prevention of human immuno-deficiency virus and other sexually transmitted infections

Participants seemed to understand that VMMC reduces the chances of HIV infection. They were also aware that medical circumcision only partially reduced the chances of infection. However, some participants did not fully understand the concept of partial HIV protection provided by VMMC.

‘When you are uncircumcised, there are infections on the collar of the penis.’ (Participant 9, male, 29 years, nursing assistant)

‘Medical circumcision is helpful to both males and females in that once the foreskin is removed there is reduced chances of infections like HPV [*human papillomavirus*], which causes cancer in females, and with males, also there is reduced incidence of other STIs [*sexually transmitted infections*] and even penile cancer.’ (Participant 13, male, 23 years, clinical associate)

‘Circumcision helps to reduce the chances of contracting HIV and other sexually transmitted diseases by 60 percent.’ (Participant 11, male, 39 years, medical doctor)

#### Traditional verses voluntary medical circumcision

Participants seemed to consider traditional circumcision in terms of the complications associated with the procedure because of the manner and context in which it is carried out. Participants in this instance seemed to understand that medical circumcision is a safer option compared with the traditional method. The understanding of healthcare workers regarding medical and traditional approaches to circumcision seems to be largely influenced by their clinical orientation to healthcare, including medical circumcision.

‘I know more about medical circumcision because that is what I have been doing here. It’s not painful, they inject you and give you medication. As far as I know, at the mountain they don’t give you any treatment. It is not safe because you can end up with infections. You can end up dead if you do not receive any treatment.’ (Participant 15, male, 22 years, clinical associate)

‘I don’t know about traditional circumcision but I know we have had so many problems. People dying, especially in the Eastern Cape, because of issues of hygiene and sterility.’ (Participant 11, male, 39 years, medical doctor)

‘Overall, medical circumcision is safe … I’m not sure if they even deal with the inner prepuce at the mountain.’ (Participant 3, male, 31 years, registered nurse)

#### Healing time versus medical circumcision time

In terms of healing time, participants agreed that it takes 6 weeks for a male to be fully healed after VMMC; healing time was well understood by participants in this regard. However, in terms of best age to circumcise, participants appeared to have a limited understanding of when VMMC can be safely and effectively performed. This was supported by the following statements:

‘It takes 6 weeks; we tell them that. We also teach them that after two weeks, maybe they will think that the wound is healing. But that does not necessarily mean that it is completely healed to engage in sexual intercourse … they need to wait for 6 weeks.’ (Participant 1, male, 27 years, registered nurse)

‘There is no specific time, but for convenience, it’s better to do them when born. But because of the anaesthesia challenges it’s better to do them when they are 8 and above because they are better able to tolerate the procedure…’ (Participant 4, male, 42 years, registered nurse)

‘For us, we start at 12 because most of the time, the younger ones less than 10 cry a lot. You cannot work properly. You cannot do the procedure properly because they will give you a little trouble. They cry and cannot keep their hands away from the procedure area. It takes even longer to finish doing them.’ (Participant 3, male, 31 years, registered nurse)

## Discussion

The purpose of this study was to examine healthcare workers’ perceptions and understanding of VMMC in KZN, South Africa. The results of this study revealed that while healthcare workers were aware of the medical rationale for VMMC, it appears that their perceptions and understanding of VMMC were primarily influenced by the cultural and religious norms that influence circumcision in the KZN context.

It was found that participants’ perceptions of VMMC in this study were centred on the nature of the procedure as a surgical intervention, one involving removal of the prepuce – a layer of skin covering the glans penis. Participants seemed to perceive this procedure to have medical benefits. This finding confirms the impact of the clinical training that health workers received regarding VMMC. Moreover, it agrees with the available evidence which suggests that medical circumcision has significant health benefits such as a reduction in HIV transmission in female-to-male sexual encounters, a reduced risk of contracting sexually transmitted infections (STIs) such as herpes, chancroid and syphilis, a reduced risk of urinary tract infections (UTIs), and a lower risk of penile cancers and carcinomas.^21,22,23^

Participants in this study revealed their perceptions of VMMC in terms of the delivery of the health service and in relation to who they thought were the ideal recipients of this service. In this regard, participants’ perceptions were influenced by their participation in and knowledge of a socio-cultural value system where circumcision is associated with traditional gender specific rites of passage. Culture and tradition are often used interchangeably in literature to depict a sense of shared values that influence human behaviour and, as a result, their response to health and ill-health.^24,25,26^ Research on the socio-cultural barriers affecting uptake VMMC among men has revealed that culture and religion can act as both barriers and drivers to the acceptability of medical circumcision by men.^12,27^ Alluding to the cultural barriers affecting VMMC among men, Nxumalo and Mchunu^28^ found that cultural and religious norms were a major influences for uptake of VMMC among Zulu men in KZN, South Africa. The present study’s finding that healthcare workers’ perceptions of VMMC are influenced by cultural dynamics is significant as it highlights the deep-rooted influence of the socio-cultural context on healthcare and health service delivery. It is therefore important that health programmes such as VMMC engage with indigenous practices and beliefs to reap the maximum benefit from such programmes.^29^ Furthermore, engagement with such practices should be directed not only to services users but also to the healthcare providers as some beliefs are held by healthcare workers and may influence how they advocate for such health programmes.

Participants’ understanding of VMMC was related to knowledge of the partial nature of protection afforded by VMMC against HIV infection and certain STIs. These findings agree with the results of previous studies that explored women’s understanding of the partial protection offered by VMMC against HIV infection.^30,31^ Healthcare workers’ demonstration of their accurate understanding of the concept of partial protection is encouraging as it shows that they will be able to relay such information to patients when mobilising for uptake to prevent men’s risky sexual health behaviour following VMMC. The provision of information to prevent risk compensation following VMMC is important for the promotion of overall positive sexual and reproductive health as post-VMMC behaviour change interventions have been shown to promote medical circumcision’s positive outcomes.^32^ The participants were aware of the nature of VMMC; most were unsure of how traditional male circumcision was performed. However, most perceived medical circumcision to be safer than traditional circumcision. Similar findings were in Malawi among circumcising and non-circumcising communities.^33^ These findings are also significant as healthcare providers are advocates for health programme implementation. In order for healthcare providers to effectively advocate, they must be aware of the details of alternative approaches. Therefore, the gap in knowledge regarding traditional approaches to male circumcision should be addressed to deepen healthcare providers understanding of the issues involving their patients.

Participants seemed to understand the VMMC healing process and the 6-week sexual abstinence period. However, in terms of VMMC performance, most participants seemed to think that younger boys would benefit more from VMMC because of the perception that younger boys are less likely to experience postoperative complications. This demonstrates a misunderstanding of VMMC on the part of healthcare workers. These findings support the belief that intensifying neonatal circumcision is preferable because it is associated with fewer medical risks.^34^ These findings represent one of the many perceptual barriers that hinder the uptake of VMMC by older males. Furthermore, the results of the current study suggest that this barrier may be perpetuated by an inherent misperception among healthcare workers. Other studies have also found that healthcare workers lack knowledge about VMMC and the need for HIV testing.^35^ Training interventions aimed at healthcare workers thus remain instrumental to facilitating the successful uptake of VMMC since demand generation activities rely on the messaging provided by healthcare workers.

## Limitations

The study was limited as a result of the small sample size and the research design used, which did not allow for generalisation of the findings. The choice of individual in-depth interviews as the sole method of data collection may have compromised the depth of findings. Nevertheless, the study findings provide an awareness of healthcare workers’ perceptions and understanding of VMMC which may help to inform training interventions that may be required by healthcare workers. This may in turn ensure that healthcare workers are better equipped for demand generation activities such as providing tailored patient messaging about VMMC.

## Conclusion

Participants’ understanding of the procedure was influenced by their culture and tradition together with general misinformation about the procedure and the biology of the penis and foreskin. The perceptions of healthcare workers also seemed to be influenced mainly by stereotypical norms of culture, tradition and the prevailing hegemonic masculinity.

The study findings provide important information on some of the impediments to the successful scale-up of VMMC. They also highlight the need for training interventions to be directed towards healthcare workers in order to address misinformation about VMMC; such interventions should consider the traditional and religious issues related to medical circumcision.
